# Dementia and Parkinson's Disease: Similar and Divergent Challenges in Providing Palliative Care

**DOI:** 10.3389/fneur.2019.00054

**Published:** 2019-03-11

**Authors:** Jenny T. van der Steen, Herma Lennaerts, Danny Hommel, Bertie Augustijn, Marieke Groot, Jeroen Hasselaar, Bastiaan R. Bloem, Raymond T. C. M. Koopmans

**Affiliations:** ^1^Department of Public Health and Primary Care, Leiden University Medical Center, Leiden, Netherlands; ^2^Department of Primary and Community Care, Radboud university medical center, Nijmegen, Netherlands; ^3^Departments of Neurology and Anesthesiology, Pain and Palliative Care, Radboud university medical center, Nijmegen, Netherlands; ^4^Department of Neurology, Donders Institute for Brain, Cognition and Behaviour, Radboud university medical center, Nijmegen, Netherlands; ^5^Groenhuysen Organisation, Roosendaal, Netherlands; ^6^Dutch Parkinson's Disease Association, Bunnik, Netherlands; ^7^Department of Anesthesiology, Pain and Palliative Care/Expertise Center for Palliative Care, Radboud university medical center, Nijmegen, Netherlands; ^8^Radboudumc Alzheimer Center, Nijmegen, Netherlands; ^9^De Waalboog “Joachim en Anna, ” Center for Specialized Geriatric Care, Nijmegen, Netherlands

**Keywords:** end of life care, hospice care, palliative care, health services, nervous system diseases

## Abstract

Dementia and Parkinson's disease are incurable neurological conditions. Patients often experience specific, complex, and varying needs along their disease trajectory. Current management typically employs a multidisciplinary team approach. Recognition is growing that this team approach should also address palliative care issues to optimize quality of life for patient and family caregivers, but it remains unclear how palliative care is best delivered. To inspire future service development and research, we compare the trajectories and conceptualization of palliative care between dementia and Parkinson's disease. Both Parkinson's disease and dementia are characterized by a protracted course, with progressive but fairly insidious development of disability. However, patients with Parkinson's disease may experience relatively stable periods initially but with time, a wide range of debilitating symptoms develops, many of which do not respond well to treatment. Eventually, dementia develops in most Parkinson patients, while motor disability develops in many dementia patients. In both diseases, symptoms such as pain, apathy, sleeping problems, falls, and a high caregiver burden are prevalent. Advance care planning has benefits in terms of being prepared before the disease progresses into a stage with communication problems or severe cognitive impairment. However, for both conditions, the protracted disease trajectories complicate conceptualization of palliative care through different stages of the disease, with pertinent questions such as when to offer what interventions pro-actively. Given the similarities and differences, we should develop palliative approaches that are partially generic and partially disease-specific. These should be integrated seamlessly with disease-specific care. Substantial research is already being performed on dementia palliative care. This may also inform the further development of palliative care for Parkinson's disease, including an evaluation of palliative interventions and services.

## Introduction

Palliative care has been developed to improve quality of life, mostly for patients with incurable cancer (1, 2). However, equity of access to palliative care involves access on the same footing for patients with other incurable diseases. This does not mean that palliative care is, or should be, the same across these diseases. On the contrary, to optimally tailor care to individuals, the contents of palliative care, and how, when and where it is delivered, can and in fact should differ between diseases.

Along the trajectory of chronic-progressive and incurable neurological diseases such as dementia or Parkinson's disease (PD), various complex needs arise, some of which are disease-specific. Palliative care promotes quality of life in the face of *any* life-threatening, or progressive, incurable illness (3, 4). To optimize it for individuals, however, a good understanding of disease-specific aspects of palliative care is helpful, i.e., a conceptualization of what palliative care entails exactly for a specific disease.

### Epidemiology of Dementia and Parkinson's Disease (PD)

Dementia and PD are both diagnosed frequently and increase mortality (5, 6). Perhaps dementia is perceived more so as a memory problem and a disease of old age, but the incidence of dementia and PD in younger age is similar. In the Netherlands, for dementia, the incidence per 1,000 person-years is 0.4 among those aged 60–64 (7), and for PD, it is 0.3 (ages 55–65) (8, 9). Dementia incidence patterns, however, show a much steeper increase with age; mounting to 27 per 1,000 person-years for those 85 and over, compared to 4 for PD over 85. In view of similar mortality (6), therefore, the prevalence of dementia in the general population is much higher than prevalence of PD (8–11). However, adjusted for age and other factors, 6-year mortality in PD is higher than in Alzheimer's dementia (6). Age adjustment is relevant also as it shows that comorbid disease may be equally prevalent for Alzheimer's–a main type of dementia–and PD across the same age groups (12).

### Comparing Trajectories and Conceptualization of Palliative Care for Dementia and PD

The two disease trajectories may overlap partly as dementia is a frequent manifestation of PD. Mild cognitive impairment may already present upon diagnosis of PD (13). Importantly, it is independently associated with lower quality of life (14). Across studies, typically about a quarter of patients with PD have dementia (15, 16), but ultimately, most develop dementia (15–17).

A clear conceptualization of palliative care in chronic-progressive diseases is important for the development of healthcare systems that facilitate the integration of a palliative approach (18). Therefore, in this article we compare the disease trajectories of dementia and PD in as far as relevant for the conceptualizations of palliative care. We do not include atypical Parkinsonian disorders such as multiple system atrophy because these warrant a special approach with earlier palliative care (19). We first provide background on where we are by describing how palliative care for dementia and PD developed.

### Palliative Care in Dementia

The first evaluated palliative care program specific to dementia was described in 1986 (20). The volume of research has grown exponential after 2000 (21, 22). There are few randomized controlled trials, and therefore, there is still little evidence on effectiveness (23, 24). However, many western countries have funded observational studies resulting in numerous publications describing patient, family and professional caregiver needs (25, 26).

Research specific to dementia is important because the course of the disease is highly variable and uncertain. Because of the progressive dementia, patients themselves often cannot remain involved in decision making. Also, health services and changes such as transfer to a hospice, do not necessarily represent optimal care for people with dementia (27). Palliative care in dementia needed a clear conceptualization, and the European Association for Palliative Care (EAPC) along with experts agreed to a distinct concept in terms of eleven domains, different from “usual” palliative care (28).

### Palliative Care in Parkinson's Disease

Palliative care for people with PD and their caregivers has progressed over the last 10 years but it is still an upcoming field. Evidence of effects is limited (17, 29, 30) but trials are underway (31). Qualitative studies on palliative care needs (32–34) and natural history studies (35–37) have indicated that the needs of people with advanced PD are complex. Awareness of the potential benefit of palliative care is growing, but we know little about useful components (17, 29). To the best of our knowledge, there is no clear conceptualization of the specifics of palliative care in PD.

## Differences and Similarities

We highlight key similarities and differences between the trajectories and perceptions of the disease, and treatment and care for people with dementia and PD based on recent literature. The comparator is a population without the disease, sometimes matched or adjusted for differences such as age or co-morbidities.

### The Disease Trajectories

With both diseases, the diagnosis may be delayed due to gradual onset with a-specific symptoms after which burdensome symptoms develop, while the disease duration is highly variable (Table 1, items a and b). Burdensome symptoms that decrease quality of life often include rather unspecific symptoms such as pain and depression. Clearly, PD is distinct from dementia as it is characterized by its motor symptoms, such as bradykinesia, rigidity, and tremor. Symptomatic treatments are available and with the right therapeutic approach, the course of PD typically includes an initially relatively stable phase.

**Table 1:** The course of dementia and Parkinson's disease: items relevant to palliative care.

**Course of the disease, items**	**Dementia**	**Parkinson's disease**
a. Diagnosis, duration, and staging	Symptoms may occur long before diagnosis of dementia. Onset is usually after age 65, but when before, duration is usually longer, and the number of life years lost is greater (5). Survival of dementia patients is highly variable between individuals and across studies with median or mean survival generally being between 3 and 12 years from diagnosis (5, 38, 39) About half may die before reaching an advanced stage of dementia when decision making and ADL functioning are severely impaired (40) and the large majority does not reach a stage in which they are totally dependent in ADLs, incontinent, bedridden, and mute (41). Definitions of severe or advanced dementia generally comprise criteria for cognition combined with criteria for behavior and function including ADL (42–44)	Prodromal symptoms, such as depression, constipation and Rapid Eye Movement (REM) sleep behavior disorder may occur long before diagnosis (45–47). Progression and survival in Parkinson's disease is highly variable, and mean duration of the disease until death ranges between 7 and 14 years (48). Patient populations are heterogeneous and complex and therefore the clinical course is variable (49). Given enough time, the disease is suggested to progress through five phases: prodromal, stable, unstable, advanced, and late-stage disease (50). However, consensus on demarcation of later stages is lacking (51, 52)
b. Symptoms (physical, psychological, social, and spiritual) and caregiver issues	Pain, agitation and shortness of breath are highly prevalent and burdensome symptoms, and pain and shortness of breath increase toward the end of life (44, 53) while agitation and other symptoms such as apathy may increase during the course of the illness in community-dwelling people (54), but may stabilize or decrease at the end of life (in nursing home populations) (44, 53) Agitation and behavioral symptoms of dementia prevalence rates vary widely but develop at some point in most people with dementia (55, 56). They add greatly to caregiver burden (57). In particular depression often compromises quality of life (58). Regarding spirituality, recent studies show that people with dementia may understand through remembrance of early life experiences (59, 60)	Diagnosis is based on motor symptoms; bradykinesia, tremor, and rigidity (61). Parkinson's disease affects physical, emotional and psychosocial aspects of life (62, 63). Compared to motor symptoms, non-motor symptoms, such as pain, depression, fatigue, psychotic phenomena are more important in terms of quality of life (64, 65) and they may be under recognized (66, 67) Cognitive deficits in Parkinson's disease may be present already at diagnosis and affect quality of life (14). There is significant impairment in executive functions such as poor planning and problem solving capacities. Up to a majority may develop dementia ultimately (15–17) Caregiver burden increases when patients reach advanced stages due to increasing disability, and also with the appearance of symptoms such as hallucinations, depression and falls (68)
c. Functioning	Progressive impairment is related to a decline in cognitive and physical functioning. There is a continuous decline in functioning, e.g., first dependency in IADL occurs, followed by ADL dependency (69). There are also various personality changes with different types of dementia; for example, apathy more commonly develops with Lewy body dementia compared with Alzheimer's disease (70, 71)	The disabling nature of Parkinson's disease increasingly hinders daily activities and social participation (72). Disease progression leads to impairments at different levels of body functions, limitations in a wide variety of ADL and IADL functioning and in a severe stage, disability, and social embarrassment occurs
d. Cause of death	Dementia is often not mentioned as a cause of death in death certificate studies, in particular when patients are younger, have mild dementia and a non-Alzheimer type of dementia (73). Immediate causes of death are often pneumonia and cardiovascular problems (73, 74) also in autopsy studies [e.g., (75, 76)]; more often so compared to people with no dementia	Parkinson's disease was not reported as the underlying or contributory cause of death on more than 53% of death certificates (77). There is a significant increase in deaths from pneumonia, dementia and other infections (78, 79)
e. Prediction of mortality	Strong predictors of mortality are functioning (ADL dependency), nutritional status or intake (80) and male gender including in acutely ill patients (81, 82). Higher age and various co-morbid conditions also relate to increased mortality. Dementia stage, however, is not a strong predictor of mortality in nursing home residents with dementia (83, 84) as a fatal pneumonia or food and fluid intake problem also occur before the advanced stage. Similarly, a late stage on the Functional Assessment Staging (FAST) scale has no predictive value (85). Of note, the available prognostic scores do not identify many at risk of death within a particular timeframe, although they can identify a reasonably sizeable group of people at low risk of mortality, within, e.g., 6 months	Strong predictors of death in people with Parkinson's disease are age, dementia, pneumonia, infections and falls (6, 79, 86–89). Other studies also found male gender, comorbidity, axial features and motor and therapy-related complications to predict mortality (90, 91). Both motor complications and non-motor symptoms were associated with mortality at 4 years in a recent study in patients with Parkinson's disease and no dementia (91)

For PD patients, loss of functional ability (item c) occurs with symptoms that are largely unresponsive to treatment speech problems, postural imbalance and cognitive deterioration; (51, 92) or with symptoms that may worsen due to treatment (psychosis, orthostatic hypotension) (51). Other reasons for functional deterioration are age-related comorbid disease (92) and under-treatment of symptoms (93, 94), which also happen with dementia. With dementia, loss of motor or functional ability often relates to progressive cognitive dysfunction.

Defining a severe or advanced stage of the disease (item a) has been recognized as important for palliative care in case of dementia (42–44). For PD, the most widely used measure to define disease stage is the Hoehn and Yahr scale (51, 52, 95). However, it selectively focuses on motor function. A recent consensus study defined key factors for diagnosing advanced PD including, for example, ADL impairment, and dementia (96).

The stage of dementia is often being perceived as relevant for palliative care although there is no consensus how exactly (28). Also, it is not a particularly strong predictor of mortality among those with moderate or severe dementia, despite sensitive measures; this may be related to uncertainty as to in what stage acute problems such as pneumonia develop or different resilience among long-term survivors (Table 1, items d and e) (74, 83, 84). Similarly, a late stage on the Functional Assessment Staging (FAST) scale has shown no predictive value (85), but practice lags behind, still promoting it for prognostication in dementia (97). ADL dependency, on the other hand, is a strong predictor of mortality in dementia (44, 80, 81). In contrast, it may not predict mortality in PD well (98). In PD, dementia or cognitive impairment independently predicts mortality (6, 35, 99).

Pneumonia is a relatively frequent cause of death in dementia and in PD (74, 78, 81). However, well-known problems in coding practice include dementia being grossly underreported on the death certificate (73), also in those with PD (78). Similarly, PD often goes unreported (79).

The overlap between PD and dementia is significant as up to a majority of patients with PD eventually develop dementia (9, 16, 17), due to spreading of Lewy bodies. Because of initial stability and uncertainty as to whether patients develop severe cognitive problems or die before, PD may be perceived as an even more protracted disease course than the dementias.

### Conceptualization of the Diseases, Needs, and Interventions

Both dementia and PD are incurable and progressive diseases with often complex problems and needs, for which tailored interventions are available (Table 2, items a–d). For dementia, experts agree that “recognizing its eventual terminal nature is the basis for anticipating future problems and an impetus to the provision of adequate palliative care” (28). Some advocate *advanced* dementia to be a terminal disease to support eligibility for palliative care. However, as about half of dementia patients never reach an advanced stage (Table 1); (40), it may be a late trigger to initiate palliative care. There is no consensus, however, at which stage palliative care in dementia should start (153, 154).

**Table 2:** Conceptualization of the disease, needs of patients and family caregivers, and interventions.

**Items about conceptualization, needs and care**	**Dementia**	**Parkinson's disease**
a. Treatment of the disease	No curative treatment is available. Some drugs such as Donepezil may improve cognition and behavior of people with Alzheimer's disease. It may not deserve labeling it as disease modifying drugs; essentially this is palliative medication because they do not slow the progression of, nor cure the disease (100)	No curative or neuroprotective agents are available. A wide range of treatment strategies are available for symptom reduction, often requiring specific Parkinson's disease expertise. Available treatments are pharmacological (e.g., dopaminergic replacement), as well as rehabilitative (e.g., physiotherapy, occupational therapy) (101)
b. Conceptualization of the disease as a terminal disease	Recognizing dementia as a terminal disease may help providing adequate palliative care (28). However, about half of family caregivers and nursing staff do not perceive it as a terminal disease or a disease you can die from (102, 103)	To our best knowledge, there is no research that examines perceptions about Parkinson's disease as a terminal disease
c. Patient's needs in an advanced stage	In advanced dementia, needs may relate to the four domains of palliative care (4), additionally, to (practical) support, and important environmental needs such as a peaceful environment (104–106). Familiarity of environment, routines and people around who know the patient and can interpret the behavior is also important (27, 107)	Prizer et al. (108) add financial needs to needs in the four domains of palliative care and find that patients and families report fewer needs in the financial and spiritual domains; on average, as these needs are more variable between individuals than needs in the other domains. Patients preferred individualized care to address psychosocial issues, adjustment to illness (particularly at diagnosis and with progression), non-motor symptom control, and advance care planning as an adjunct to usual care (109) and it is also perceived as a social need (108). Patients and family caregivers in Canada found that they are not receiving enough information about diagnosis and prognosis (33)
d. Interventions to address needs in particular in an advanced stage	Non-pharmacological treatment of symptoms such as agitation is first choice (28, 100). Because symptoms are easily missed and causes are not always easily identified, systematic assessment and treatment (such as in the stepwise STA OP! or STI intervention) is needed (110). Also regular special programs (such as Namaste Care) (111) to connect with people in the advanced stage in a peaceful atmosphere are needed when they cannot participate anymore in regular activities such as those offered in nursing homes Familiar rituals and music may be recognized until late stages of the dementia and therefore it is important to know religion or spiritual orientation (28, 59). Furthermore, spiritual, and faith practice may help cope with the disease, to find meaning in life, and they relate to wellbeing (59, 60)	Interventions are typically multifaceted and require specialist knowledge, therefore intervention programs have focused on enhancement of multidisciplinary collaboration and education of professionals (e.g., the expert network of ParkinsonNet). Evidence of effectiveness is becoming available, for example occupation therapy, physiotherapy and integrated multidisciplinary care (112–114). An international guideline in palliative care for people with PD includes recommendations for specific late-stage problems (115). However, no study has focused specifically on addressing palliative care needs
e. Assessment tools	Tools specific to dementia are needed for the assessment of pain, distress and behavior (116, 117). There are over 30 pain observation tools available (116) and there are also inventories for multiple symptoms that, in contrast, include pain as a single item such as the Integrated Palliative care Outcome Scale for Dementia (117)	There are symptom assessment tools adapted from generic tools that could identify specific palliative care needs in PD, such as the Palliative care Outcome Scale Parkinson disease (POS-PP) and the Edmonton Symptom Assessment System Parkinson's Disease (ESAS-PD) (49, 118, 119)
f. Place of death and continuity of care	In western countries, people with dementia in a moderate or severe stage are often admitted to a residential or nursing home which is also the most common site of death in most western countries. However, comparing several studies, home death was more common in Southern European countries and Mexico, and hospital death in (developed) Asian countries (120). Japan, for example, refers patients with dementia with behavioral problems to psychiatric inpatient care and people may die there (121). Continuity of care in the last year of life with dementia is problematic also in western countries including in the US and Finland (122, 123)	A substantial proportion of deaths with PD occur in a hospital, although there is wide variation between countries. A study in 11 countries showed that hospital death was most prevalent in France, Hungary and South Korea, whereas nursing home death was most common in New Zealand, Belgium, USA, Canada and Czech Republic; and home death in Mexico, Italy and Spain (124). Patients with PD had more physician consultations and more emergency department visits per year compared to patients without PD (125)
g. Communication, decision making and the patient's perspective	Due to increasing cognitive problems, communication with people with dementia changes. Apprehension of risk changes and health numeracy decreases; patients are often not involved in treatment decisions (126, 127). Shared decision making models need an extended preparatory phase to first examine perceptions of the need for a decision (128) Palliative care or comfort care is often, but not always preferred for nursing home residents with dementia from the perspective of patient and family caregiver, in different countries (107, 129, 130). In a hypothetical situation of advanced dementia, most older people would opt for comfort care in a study in rural areas (131). Patient advocacy organizations have issued recommendations for end-of-life and palliative care in the advanced stages of dementia (132–134)	Dissatisfactory communication with professionals is one of the most common complaints of patients with PD (109, 135). Treatment of the disease is mainly driven by the clinician (136) In a hypothetical end-of-life situation the majority of proxies of patients with advanced PD would choose comfort care as the goal of treatment (137). A US patient and family caregiver council advocates palliative care to be available from diagnosis (138)
h. Advance care planning (ACP)	ACP often does not start until the late stage when the patient cannot be involved anymore. It is not always clear whose responsibility it is, there are multiple barriers including patients rather living by the day, and there may be discontinuity of information with a change of setting of care (126). However, in view of the cognitive decline, ACP is preferably started early (28). There is also evidence of effectiveness to increase advance decision making, family satisfaction with it, and other outcomes (23, 139, 140)	Many patients want information on prognosis early in the disease. Patients' preferences regarding communication and timing of end-of-life discussions vary (141). A full ACP process may be perceived as lowering mood in an early stage the disease, and should therefore be tailored to the stage of the disease and individual preferences (135). Only a few patients with PD who died in a UK hospital had had end-of-life care discussions which were documented (142)
i. Care for families	Family caregivers usually need support and care themselves including support in proxy decision making, long before the dying phase (28). Caring for a person with dementia is often highly burdensome especially when behavioral symptoms such as agitation and sleep disturbance develop (143). In addition to higher caregiver burden, there are fewer positive caregiving experiences, even at the end of life (144) Pre-grief often occurs with the decline of the patient, especially among spouses (145). Psychosocial interventions may decrease pre-grief (146)	Family dynamic change (30, 68, 147–149), loss of autonomy, economic strain and social isolation are part of the caregiver burden Pre-death grief was a significant finding in family caregivers of patients with advanced PD and was associated with a patient's cognitive decline (150). In a review (151) of 30 studies about interventions to support caregivers only one psychosocial intervention was shown to significantly decrease psychosocial problems and need for help (152). Interventions that embrace psycho-educational skills such as problem solving, goal setting and cognitive restructuring can bring benefit (29)

For PD there are no curative treatments either, but the success of dopaminergic replacement therapy and deep brain stimulation has enabled the majority of patients to live independently with a relatively low symptom burden for the first 10 years after diagnosis-when they live up to a decade (48). This may contribute to PD generally not being recognized as an illness for which a palliative approach may be helpful (155, 156). A US patient and caregivers council recommends palliative care to be available from diagnosis of PD (138). This is also the ideal of the European Parkinson's Disease Association (EPDA) (157) although they emphasize that when to start palliative care is an individual decision.

Patients with dementia may have a number of needs in the four domains of palliative care (physical, psychological, social and spiritual) in addition to specific needs for a peaceful, familiar environment, and practical support (104–106). Typically, complex, multifaceted interventions could address needs. Psychosocial needs may be pronounced in young-onset dementia (onset under age 65) (158, 159). Patient advocacy organizations recognize the importance of high-quality end-of-life and palliative care in the advanced stages (132–134).

To measure symptom burden, specific tools are available (Table 2, item e). For dementia, these typically involve proxy (caregiver) report. Quality of care and dying assessment tools specifically developed for dementia show the best psychometric properties (129). For PD, there are adapted versions of generic tools (118, 160). Regardless, effective use of tools and implementation of complex interventions requires multidisciplinary communication and team work (161).

Place of death varies by country (Table 2, item f). Patterns are similar for dementia (120, 121) and PD (124), with dying in nursing homes being common in many western countries except Southern European countries with more frequent home death, while hospital death is more common in Asian countries, France and Hungary. In the UK, a trend of decreasing hospital death and increasing nursing home death has been observed in dementia (162). However, continuity of care may be problematic across countries, with nursing home or hospital admissions at the end of life in people with dementia and PD (122–124, 163). Also, with PD, specific knowledge of the disease is often suboptimal among nursing staff in nursing homes, while upon admission, neurologists often stop seeing patients with PD, with communication of neurologists with primary care being suboptimal too (93, 94).

“Person-centered care, communication and shared decision making” was among the most important domains of palliative care in dementia according to experts around the world, and it was prioritized for research (28). Advance care planning (ACP) is a special form of ongoing communication about preferred future health care (Table 2, items g and h). Researchers and policy makers are increasingly interested in researching and implementing ACP, and some beneficial effects have been documented in dementia (23, 139, 140). However, there are numerous barriers such as patient and family expecting the physician to start it while physicians may not prioritize such anticipatory care. Also, while many appreciate discussions, not all would like to decide about future treatment (126, 164). Similar barriers might exist in PD (141). In a synthesis of studies, interview studies have shown physicians to be hesitant in discussing progression of the disease in early disease stages as they fear to diminish hope (17). Also patients indicate a conflicted need as they reported both a wish for more information on disease progression and death, and fear to receive the information (“an information tension”) (17).

Family caregivers usually have information and support needs in regards to proxy decision making and how to best care for their loved one (28, 165) (Table 2, item i). They may experience a magnitude of stress in caregiving and pre-grief in dementia (145) and PD caregiving (150). In dementia, anticipatory grief in response to compounded serial losses is common, and stress in caregiving preceding physical death may be equal to or greater than stress in bereavement. Roland et al. (166) found caregiving experiences and stressors to be similar between caregivers care for a patient with dementia, PD, and PD and dementia.

### Disease-Specific Palliative Care and Practice

Disease-specific palliative care is needed; services and tools taken uncritically from cancer palliative care have shown to not fit well with dementia and require adaptation or even redevelopment from scratch (27, 167, 168). Palliative care specialists, however, may not know enough about the specifics of dementia and PD. In addition to suboptimal access to palliative care (17) access *to disease-specific* multidisciplinary care for PD may be suboptimal (93, 169, 170).

Clearly, better integration of disease-specific and palliative care expertise is needed. To establish dementia-specific palliative care, the EAPC therefore recommends collaboration between disease-specific (dementia) and palliative care (28). In the UK there are initiatives for outreach with specialist dementia palliative care to support the familiar care team (27, 171, 172).

Regarding PD, the provision of palliative care is widely advocated (17, 29, 32, 173). A special task force of the International Parkinson & Movement Disease Society is dedicated to improving palliative care in PD (29, 174). A mapping exercise in the UK showed service provision to vary across regions, and services for PD were not well-integrated with palliative care (175). There are some patchy examples of integration of expertise from a palliative care department with a neurology department (176). Patients and family caregivers found they lacked knowledge about palliative care services. Only few patients received care from a palliative care service and coordination of care was poor (33, 147, 148). The need for palliative care, including early in the disease trajectory, has been emphasized by a collaborative statement from the EAPC and the European Academy of Neurology (EAN) (177).

Among dementia care specialists, providing palliative care early is controversial (154, 171). Soon after diagnosis, palliative care can start in the form of ACP if patient and family caregiver are willing to talk about the future (28). Waiting until an advanced stage means many will never receive palliative care, mortality having been predicted inaccurately so patients die well before palliative care issues could be addressed. Establishing criteria to restrict access to US hospice care to those closest to the end of life has been subject of considerable research [e.g., (82, 85)]. Prediction research consistently shows we can identify those likely to survive accurately, but not, or very few of those likely to die. Needs may differ with more advanced dementia though, and ideally, a needs-based approach is adopted (176). Similarly, for people with PD, triggers for palliative care (98) and access to US hospice care have been sought using a mortality prediction approach (178). For example, a BMI less than 18.5, accelerated weight loss and reduction of dopaminergic medications was suggested for referral to US hospice care (178).

There is a lack of awareness about palliative care being applicable to dementia both among the general public and health care professionals, and this is perceived as a major barrier to improve palliative care, for example by Dutch and British physicians (179). Nursing staff may feel that they lack competencies to deliver high-quality palliative dementia care (Bolt et al., under review). Also, in PD, professionals may feel uncertain about the palliative care they deliver and often experience a lack of education and competence in this field (155, 156, 180, 181).

## Conclusion

Substantial research is being performed on dementia palliative care. Much has happened since early descriptive research in dementia compared symptoms and treatment with cancer [e.g., (182)], and introduced a hospice model of care (20). The research has culminated into a clearer definition of what palliative care should entail with dementia and into some understanding of its effects. Comparisons with other diseases are now available regarding a variety of aspects (e.g., a higher caregiver burden compared with cancer (183), symptoms compared with various other chronic-progressive diseases (184), or specific problems described in subgroups with both dementia and cancer (185–188). Nevertheless, it is still unclear what is important at what stage and how to best incorporate individual preferences, for example regarding discussions about future care.

PD follows an even more protracted course which complicates a clear definition and there is no agreed-upon, evidence-based, disease-specific conceptualization of palliative care. Even more varied multidisciplinary expertise may be needed including also dementia care expertise (in addition to PD disease-specific care, palliative care and generic long-term care for older people). More specific tools may also be needed, for example, application of pain observation tools in PD dementia should consider that facial expressions indicating pain are distinct [e.g., less eye narrowing but similar upper lip raising (189)], to avoid possible underreporting in Parkinson's disease compared with Alzheimer's disease (190).

The combining of various expertise requires clear roles and inter-professional collaboration which is challenging in the face of uncertain disease trajectories (191). However, integration of palliative care has shown to improve process outcomes and patient and caregiver outcomes in cancer and chronic-progressive disease (192). Integration should take place at the clinical (patient) level but also, for example, through relationships between professionals and between organizations and in the wider system (193). For this, multidisciplinary networking and teams sharing expertise is important, supported more formally by shared guidelines and pathways (194).

Sawatzky et al. (18) describe three reasons how a clear conceptualization of palliative care in chronic-progressive disease may be helpful: (1) earlier recognition (“upstream”) of needs, (2) to promote adaptation of palliative care knowledge and expertise for unique disease profiles, (3) to operationalize a palliative approach through integration into systems and models of care that do not specialize in palliative care. Such conceptualization is promoted by looking at similarities with other diseases with more established palliative care models, but also taking a closer look at divergences, such as the initial stable phase in PD and how this should affect palliative care services. It raises the question whether ACP is an integral part of palliative care, or could precede it, also for dementia considering ambiguity around early palliative care. This resonates with recommendations of Temel et al. (195) for “early” palliative care to depend on the type of cancer; with low symptom burden, waiting until a change in health status or emergency room admission may be a reasonable approach.

For palliative dementia care, it has been helpful to also consider how it differs from “usual” dementia care, for example, by a highly proactive approach. Such understanding of what needs to be changed in practice facilitates the integration of a palliative approach in dementia care so that ultimately, the integrated care becomes the standard (196). With Kluger et al. (29), we believe that palliative care in PD will benefit from a clearer conceptualization.

Although probably not directly suitable as entry criteria for palliative care, research on prognostic factors in PD may be helpful. For example, ADL dependency strongly predicts mortality in older people and in dementia (80, 81, 85), probably covering cognitive and physical impairments and other risk factors. In contrast, it may not predict mortality in PD well (98), perhaps because motor function declines early; some dependency therefore occurs earlier than in dementia. Further, we agree that more work is needed regarding assessment of needs–including spiritual needs, development of assessment and educational tools and interventions for patient and caregiver support (29, 108).

Although we could not compare directly, we hope that our review contributes to an emerging understanding as to what elements of palliative care with neurological conditions are disease-specific and which are more general. We acknowledge that the brevity of the review did not allow more depth regarding aspects of the disease and care in different types of the diseases or forms of parkinsonism which need further research. More could be written about possible implications such as how to organize disease-specific palliative care in a cost-effective manner. We recognize a need for basic guidance for clinical practice which we offer in Box 1, awaiting high-quality trials and other research we need to build refined evidence-based practices that optimally serve individuals with neurological disease.

Box 1 Basic recommendations for practice of palliative care based on similarities and divergences between progressive neurological diseases and available evidence^*^.

1. Do not wait with bringing palliative care to the table until a late or terminal stage of the disease. Although it seems an obvious and safe choice to limit to a late stage, it may be too late to involve the patient or to implement a palliative care treatment plan. With PD, there is often opportunity to speak about palliative care when cognitive problems are still mild or absent. With dementia, it is difficult to predict who will die already before the late stage, while many will, with unmet palliative care needs. Therefore, discussion of palliative care before a moderate stage is recommended.
2. Improving awareness, among all involved, of the progressive course of the disease supports a shared understanding of the disease, implications for death and dying and what it means for the individuals involved. This will be helpful in identifying and addressing palliative care needs.
3. Common causes of hospital admissions include pneumonia, sepsis, and falls. Physicians could discuss these scenarios as a starting point to establish patients' views and preferences regarding invasive therapies and the benefits of a palliative care approach.
4. Elicit preferences of patient and family and the preferred style with regard to talking about future care and end-of-life scenarios. Address any information needs, and a step-wise approach with discussions continued later on may avoid feelings of being overwhelmed.
5. The course of the disease is uncertain, whereas *change* is. All members of a care team should help identify and discuss subtle changes in symptom and caregiver burden early.
6. Palliative care is an approach in which intervening is still possible even if active treatment of the disease or its complications is not possible or when other treatment is being withheld. It can be a potent adjunct to usual care but it should be well-integrated, also at a system level. As opposed to being *uni*form, straightforward, hassle free fix, “*multi*” is the important term in this: *mult*ifaceted interventions targeted to the individual in a context of *multi*disciplinary collaboration between generalists and disease and palliative care specialists.
7. Tools to identify needs and a change in the patient's condition (physical, psychosocial, spiritual, caregiver needs) should be sufficiently *specific* to the disease while a context or setting *specific* system should be in place to support its continued use (for example, a systematic approach to managing pain, behavioral symptoms, autonomic dysfunction, sleep dysfunction or motor fluctuations/dyskinesias sustainably implemented in long-term or acute care).
8. Pre-grief with progressive decline of the patient and prolonged social isolation are common. Psychosocial support is needed in different phases to empower patients and family caregivers to cope with both chronic stressors and crises.

^*^We inferred this general and more disease-specific guidance from the items on course of the diseases and conceptualizations in Tables 1, 2, acknowledging that these are only a couple of key recommendations and that evidence is limited. Also, this guidance should be refined and fit the local context when implemented. For more detailed guidance for clinical practice, we refer to the recommendations as part of the European Association for Palliative Care (EAPC) dementia white paper (28) and guidelines from the Irish Palliative Care in Parkinson's Disease Group (115).

## Author Contributions

JS, HL, and DH: manuscript development, manuscript writing, and manuscript authorization; BA, MG, JH, BB, RK: manuscript writing and manuscript authorization.

### Conflict of Interest Statement

The authors declare that the research was conducted in the absence of any commercial or financial relationships that could be construed as a potential conflict of interest.
